# SDM Training Modules for Health and Social Care Professionals in Israel

**DOI:** 10.3389/fpsyt.2021.679036

**Published:** 2021-09-27

**Authors:** Carolyn Gutman, Ayala Cohen, Dorit Redlich Amirav

**Affiliations:** ^1^Department of Social Work, Tel Hai College, Upper Galilee, Israel; ^2^Department of Occupational Therapy, Sackler School of Medicine, Tel Aviv University, Tel Aviv, Israel

**Keywords:** shared decision making, attitudes, training, clinical practice, lived experience, non-medical professions, allied health and social care professions

## Abstract

While the strategy of Shared Decision Making (SDM) originated in the medical field and was later adopted into the mental health arena, little attention has been paid to practice in the broader fields of the allied health and social care professions. These professions are grounded in the recognition of a need for practice that reflects the partnership and collaboration of the professional and the service user working together to further the health and well-being of the user. A pilot training module was developed to introduce and support students in their journey from exposure to the co-production ideology and the SDM strategy into clinical practice in the allied health and social care professions. The aim of the present article is to describe the students' experiences while learning about SDM and their use of this knowledge in their field practice in Israel. The students' experiences highlighted the complexity of integrating SDM into practice both at the individual student level as well as the macro environment. Moreover, it pointed to the need to further develop this co-production paradigm and the SDM strategy into the education of the allied health and social care professions.

“*I felt a lot of things were done to me rather than with me”.*

*(Adelphi Research UK, 2018 p.11*. *https://www.adelphigroup.com/adelphi-research-uk/**)*

## Background

The present article focuses on the need for integrating the central professional concept of partnership into the clinical practice of allied health and social care professionals. We argue for investing in the training of these professionals on Shared Decision Making (SDM) as a tool to support this professional value system. Framed in this context, and based on previous SDM training principles, the introduction of a pilot SDM training module into two academic programs for allied health and social care in Israel is described.

Historically, the work of professionals in the health and social care fields (such as social workers, occupational therapists and nurses) is grounded in the core values of self- determination and client-centered practice. This translates into the workers' collaborating with their clients to ensure their active partnership in the process of effecting change in their lives (1). Accordingly, these principles are reflected in the different professions' codes of ethics (2, 3).

However, the allied health and social care professions are often conducted in host settings such as hospitals, schools or care homes where value discrepancies between “hosts” and “guests” can impinge on professional practice (4). Here the traditional, yet still dominant, medical model of practice that rules these settings by focusing on the clients' impaired functioning and dependency, results in the continuation of a paternalistic hierarchy between the participants in the helping relationship. Thus, while our professions charge us to work in partnership with our clients, professional practice often reflects a different reality, resulting in clients characterizing their hierarchical relationships with us as experiences of oppression with the delegitimization of their knowledge (5) creating a sense of powerlessness, especially in areas of control over resources, legitimization of knowledge, assessments, and determination of needs (6, 7).

The era of civil rights activism in the 1960s in western countries such as the US and UK created the impetus for questioning this traditional medical model of practice and creating a new discourse, emerging in particular from the disability rights movement (8).

Today, this new discourse, grounded in principles of participation and partnership, have become buzzwords in the allied health and social care professional literature. For example, it is suggested that the term partnership incorporates concepts of equality and equal power sharing between workers and clients, recognizing that each brings areas of strength and expertise and each enjoys rights and choices (9). Furthermore, work in the mental health field suggests that providing people with more choice within the context of a strong therapeutic relationship appears to predict better user outcomes (10).

Therefore, it seemed important that ideas of client partnership be expanded and more fully incorporated into the professional training and practice with people needing our professional services.

The Shared Decision Making process (SDM) was developed first in the context of terminal physical illness (for example cancer), at those significant intersections where decisions concerning intervention (11), consultations, and primary care are taken (9). The earliest mention of SDM was in 1982 (12), but the idea draws on and deepens the principles of patient centered care (11, 12).

Policies to promote shared decision making have become visible in countries such as the United States, Canada, and the United Kingdom (13). In Israel, SDM has also been introduced into the health field (14, 15) and more recently into areas of mental health and appears to be showing promise as a positive contribution to clinical practice. SDM is increasingly well-established in the medical literature (16) with a growing evidence base in mental health. Here, SDM was used initially, as a response to the well-documented difficulties in decision making regarding psychiatric medications (17). A recent review on SDM in mental health (18) reported that SDM was aligned with the core principles of user involvement and participation, person-centered care, and personal recovery, principles which are increasingly becoming appreciated in the Western world (19).

Despite a growing body of evidence in the mental health field (20) it has not explicitly adopted structured “shared decision making” (21) nor prepared users and providers for its use (22).

Moreover, it has increasingly been appreciated that clients/users are cared for not only by physicians. It is therefore important that SDM be incorporated into the practice of allied and social care professionals. In the field of social work, there are voices claiming that there is a need for SDM to be promoted as a way to further client participation in policy practice (23). Levin (23, 24) had commented that while social work professionals view SDM as representing ideas of hope, change, identity and choice, they also express frustration that the rhetoric of client participation is strongly challenged by the clients' characteristics such as their degree of knowledge and responsibility and assertiveness as well as by the challenges resulting from the disparity between the principles of SDM and the professional frameworks where they are to be implemented.

Most of the SDM literature focuses on work undertaken in the medical field and little data exists about the implementation of SDM in the allied health and social care professions. Bringing about change in these fields is complex. Drawing from research in the aligning field of social policy, findings on the implementation of a reform in social services in Israel with regard to child protection and treatment, Alfandari (25) maintained that adoption of a good reform is not enough for it to be implemented. Policy makers cannot suffice with the development of good ideas and plans that are not professionally accepted. They are required to make well-defined efforts for building and anchoring a system and an action force that will create the conditions that will allow implementation in the field.

Therefore, in order to bring about more widespread use of SDM, changes have to occur not only at the organizational/policy levels but also in the value system that frames the professional's clinical practice. This begs the need to look at professional training, both for established practitioners as well as for students and neophyte professionals.

The training of professionals in SDM has burgeoned in recent years, focusing mainly on the health professions, and aimed mostly at physicians and nurses, with the majority being developed and conducted in the US and the UK (26, 27). These trainings vary between face-to-face workshops and courses to internet based models, varying in length, aimed at particular professions and even particular fields within the profession. There is however a shortage of trainings available for the allied health and social care professions (26).

An important development in the field of SDM training other than for physicians has been recently developed in the UK (28) with SDM training programs for social workers, nurses, occupational therapists, and others, in the mental health field. This article will describe our experience with developing and conducting a pilot SDM training program in Israel, which specifically targeted a wide audience of a variety of allied health and social care professions in different settings.

### The Training Module

The training module in Shared Decision Making (SDM) was based on the previously mentioned UK model (28) that was originally designed for both mental health professionals and service users in the UK. We adapted this model to expand its use from mental health to other fields in the health and social care professions in Israel.

### Content

The current module in Shared Decision Making (SDM) was based on SDM training principles for both mental health professionals and service users. These principles affirm adoption of SDM as a process rather than an outcome that demonstrates co-production within the partnership where the user is recognized as the expert by experience; and, the importance of encouraging service users to shape their preferences and to express these in ways that can be heard by professionals.

Additionally, this training module was designed to introduce the students to the values and practice of SDM, thereby opening up opportunities for its adoption in the everyday practice within the health and social care professions.

The core content of the training module comprised the following topics:

- overview and rationale of SDM history, key components of SDM and definitions
- barriers and facilitators of SDM
- recognition of power imbalances in the professional relationship
- the components of collaborative relationships
- the contributions of decision aids to the SDM process
- identification of potential ethical dilemmas in professional decision making processes

This content, largely based on current experience on SDM in the medical and mental health fields, was delivered in this pilot training using a range of interactive methods that included:

- slide presentations with video clips from different countries
- especially developed video material with a variety of clinical scenarios
- small group exercises
- handouts and referrals to resource materials
- general group discussions
- guest speakers involved in local SDM projects

The integration of these multiple teaching methods provided the setting for introducing the implementation of SDM into the students' practice as an integral part of their professional value system.

The pilot SDM training module was conducted face-to-face at each of two higher education sites: the first with graduate student practitioners in the OT department at Tel-Aviv University and the second at Tel Hai College in the Social Work department with undergraduate social work students.

The module comprised three full day workshops for a total of 24 h.

### Participants

The participants in the Tel Hai training module were 22 final year undergraduate social work students (18 female and four male) who were part of the Social Policy track in the undergraduate program and who were enrolled in the track's Research Seminar. The training for the Tel-Aviv students was conducted in the Occupational Therapy department with 18 graduate students (16 female and two male) from a variety of disciplines: Physiotherapists, occupational therapists, psychologists, speech therapists, and nursing who were currently working in a variety of social and health settings.

### Evaluation

The data for the pilot study was drawn from quantitative and qualitative sources:

#### Quantitative Data

A feedback questionnaire was developed by the training team and was administered at the close of the training. The questionnaire used a 5-point Likert scale and addressed the students' views on the training process and the relevance of SDM to their clinical practice. Descriptive statistics were used to analyze the responses to the Likert scale.

#### Qualitative Data

1. A short open-ended questionnaire at the outset of the module looked at the students' knowledge of SDM, where they had heard about it, it's perceived relevance to their clinical practice and their learning expectations.
2. A written assignment was given for the students to complete between the second and third workshops. Here they described a planned shared decision making intervention and implemented it. They were then asked to reflect on the barriers and facilitators of the SDM process from the material that arose throughout the different stages of their preparation and practice and that of their clients'.
3. Transcriptions of notes taken by the team members during the different stages of the module. For example, material was drawn from the third workshop when the students presented their experiences with the assignment and shared their insights with the group. They also discussed their examples of using SDM with different intervention modalities (individual, family, group and community levels) as well as with diverse population groups (the elderly, children and youth).

The team's notes during workshop discussions throughout the module together with the written material obtained from the practice assignment were transcribed. Then, the qualitative data was analyzed according to a modified method of qualitative content analysis (29) in order to elicit meanings and insights from the text and identify major themes.

## Results

### Quantitative Data

Replies to the Likert scale administered at the end of the module on the training process and the perceived relevance of SDM to clinical practice provided further information. Response rate for this form was 66% (*n* = 27).

Interestingly the overwhelming majority of students (78%) from both sites underscored the importance of using SDM in their practice, and they also positively rated the content of the module as providing them with sufficient practice and feedback (see Table 1).

**Table 1:** Quantitative summary of the module.

		**0**	**1**	**2**	**3**	**4**	**5**
		**N/A**	**Strongly disagree**	**Disagree**	**Neutral**	**Agree**	**Strongly agree**
1.	The workshop highlighted for me the importance of using SDM					6	21
2.	I received sufficient information about the overall goals of the module		1		7	10	9
3.	These workshops matched my expectations		3	7	6	7	4
4.	The content of the workshops is relevant to my work		1	2	3	7	14
5.	The goals of each workshop were clear to me		1	3	8	8	7
6.	The activities in the workshops stimulated my curiosity		2	7	8	2	8
7.	The activities in the workshops provided me with sufficient practice and feedback		1	3	8	10	5
8.	The degree of difficulty of the workshops was good				9	11	7
9.	The pace of the workshops was right for me		5	5	6	7	4
10.	I reached the goals of the module		1	3	4	9	10

### Qualitative Data

The responses to the open-ended questions of the feedback questionnaire prior to the first workshop highlighted that while the overwhelming majority of the students' recognized the importance of SDM in their practice, they also acknowledged that they did not have the tools to implement it. On the question about the use of SDM with different user populations, all the students claimed that SDM could be implemented with all types of users and their families.

Following the training module, most of the students acknowledged many of the components of SDM and recognized the centrality of the users' place in the process. However, some of them referred to the importance of involving the user in the decision making process rather than genuinely creating a partnership with them. The content analysis based on the multiple sources of qualitative data yielded two main themes: the first addresses the challenges of moving from theory to practice (“Knowing and Doing” SDM: bridging the gap). The second theme focuses on the role of the practice settings (“We don't do it here”: pressures and excuses). Each theme is described below with illustrative comments.

### “Knowing and Doing” SDM: Bridging the Gap

The first theme relates to the gap that exists between the students' knowledge of the value systems driving their professions, in this case, user involvement and partnership in the helping relationship, and that of its practice. The students' understanding of the theoretical underpinnings of the allied health and social care professions did not serve them to realize true user involvement. In line with their theoretical knowledge, at the outset of the module, the students expressed enthusiasm and support regarding the value and need for partnering with users in making decisions about their lives.

“*I believe this will be suitable for work with a lot of our client groups, and will benefit the development of cooperation with the client.”*

However, during early discussions, some of the students expressed their disappointment that, in their view, the training content did not enrich their existing knowledge, as they had already previously learned about user involvement and user participation in their studies.

“[T]*his isn't new, we have heard this all before – last year in our practice course and field work ….”*

Yet following the completion of the module, these students acknowledged that, despite previously learning the value system surrounding SDM in their studies, they had been limited in their knowledge and skills to implement it into their practice.

“Here *I learned how to do SDM … before I knew about it cognitively and rationally and now, I have grasped the importance from an emotional place.”*

“*I learned ways to do it [SDM] from examples where it is good.”*

The gap between “knowing and doing” is clearly reflected by this student who sheepishly admitted,

“*It is one thing to know about SDM and something different to do it. At the beginning I didn't understand what was different between what we had already learned throughout our studies but toward the end of the module I understood – the training really sharpened the point.”*

As part of the learning process, the students now needed to reflect on the importance of alternative knowledge sources such as the value of experiential knowledge. Previously the students' knowledge base had prepared them to attribute far more to listening to their own expertise than to users' voices and experiences. This recognition was heard through the students' voices throughout the module,

“*It is important to involve the client in a transparent way about decisions and changes that are related to him and his treatment – it is critical as a base for trust that is essential for the success of the intervention.”*

“*Until my participation in the course, I gave more importance to ‘expert knowledge'. However, my way of thinking has changed greatly regarding the ‘expert from experience' and I would very much like to share what I learned in the course with my patients and not to make any decisions for them. Their decision is central even if it is very different from the way I perceive things.”*

“*During the assignment, I noticed that I don't really listen to users. I rely mostly on my expertise.”*

Some students addressed their difficulty in recognizing the power hierarchies that were inherent in their professional interactions with users.

“*Although sometimes I feel that I know what the right thing is for the child, we need to step back and llisten to our clients – their voice is what is important – the client is the expert”*.

“*One time while I was listening carefully to the words the mother said, I also tried to understand the words she did not say - the distress she was in, and her difficulty in making the decision - she wanted the best for her child, but it was difficult for her to decide which of these choices was best for him. I realized that in the next meeting it would be important for me to strengthen her ability to choose - not only on the technical side, but also on the emotional side.”*

“*I tried to use it [SDM] with my clients, and then I started to realize that there are a lot of things that I want for them and the training made me see that I need to give more space for what they want…”*

“*The client knows what is best for her… everything needs to be out there on the table … our solutions as professionals are mistaken and undermine the autonomy, the freedom, the independence and the responsibility of the client.”*

### “We Don't Do It Here”: Pressures and Excuses

The second theme relates to the role of the practice settings in the learning experience. While this is a pilot study of delivering a training model into the allied health and social care professions, the findings illustrate how frequently professional practice is shaped by the approach of the host profession in the organization namely, the medical model. The following description, through the students' voices, draws attention to the barriers and challenges they faced when introducing SDM into their practice settings. These barriers were recognized by the students to occur in two areas in the care setting: the first is integral to the organizational structure and the second in the cultural context.

#### The Organizational Structure

Firstly, the barriers and challenges that were perceived by the students relate to the different types of care settings, whether it be a hospital, a school or a prison, grounded in bureaucratic structures such as in the development of practice protocols.

As one student working in a prison complained that “…*where I work is not in line with this approach [SDM] and I feel the gap in the field.”*

One student pointed out the difficulties of the SDM process as being time consuming,

“*Sometimes it is hard to give the clients their autonomy. Particularly in a system where an important focus of our work is placed on time constraints.”*

The same student went on and pointed to the reporting procedures that did not recognize the time needed to carry out the SDM process. She admitted that,

“*Even when I asked my supervisor how to report the intervention process, she said it was too long to fit the computer's definitions.”*

“*Sometimes it is hard to stay with the client's pace and wishes as the system is rigid and wants to speed up the client's discharge process from hospital. I feel like we as professionals even if we want to stay attuned to the patients we are on our own and there is no support from the hospital for being with the clients.”*

#### The Cultural Context

The second area relates to the cultural environment surrounding the practice settings as characterized by the workers and users themselves as reflected in the students' words. Here the cultural context represents an environment that is not open to new value systems and the accompanying discourses, such as co-production or partnership and SDM with users. This unfamiliarity hindered open communication between the students and their professional colleagues and supervisors.

“*It's a method that hasn't been adopted enough because there are people who don't believe in it and find it difficult to use in certain areas, such as the prison service”*

Furthermore, the students also reported on their difficulty in introducing the principles of SDM into their relationship with clients. Specifically, they pointed to cases of users who could not access needed resources to be partners in the SDM process, whether in the form of limited knowledge about locating information and other material resources or in the form of personal characteristics such as passiveness or cognitive issues.

As one student related,

“*[C]hanging the balance of power between a patient and a provider is not so easy especially in hospitals and other systems”*.

Another example of the difficulty in engaging users into the SDM process was voiced by a mature student who told of her clients' unease with questioning professional judgements. For instance when facing a panel.

“One of my patients had to face a room full of people… professionals who discussed my client's life between themselves. They decided that she should go to a community hostel as the best option. She told me afterwards that because all the professionals were sitting together, she didn't really feel she could say anything.”

“*Our clients don't see themselves as the experts and there needs to be a lot of work to make them feel empowered enough to be able to take on their share in the responsibility for the joint work process”*

“*For me the difficulty was integrating active listening, exploration of different possibilities, and letting the client to take more responsibility on their journey, especially with my clients [with cognitive disabilities]”*.

“*Shared Decision making is not a one-time event … and what do you do with young children?”*

## Discussion

The present article follows the delivery of a pilot SDM training module into the students' curricula in the allied health and social care professions at two sites in Israel. It explores the potential for expanding SDM integration and training beyond the medical field for these professions. We described a pilot training module for these fields in two different sites with students from a variety of allied health and social care professions such as social work, occupational therapy, speech therapy and psychology. These students reflected their learning experiences throughout the module with practice experiences from fields such as child welfare, prisons, schools, residential care, and rehabilitation.

The following section is a discussion of the main themes that were identified from the students' voices throughout the training module together with thoughts on the practical implications. The section will conclude with lessons learned for strengthening the implementation of SDM into professional practice in the allied health and social care fields.

### “Knowing and Doing” SDM: Bridging the Gap

At the outset of the module, the students expressed enthusiasm and support regarding the focus of user's participation in making decisions about their lives. In the literature on SDM, medical professionals also value its place in practice (18). However, the key message from our students related to the gap that exists between the values and content of the SDM training module and the reality of the dominant medical discourse that they encounter throughout their professional education and also later in the practice arena. This gap echoes the view of Kienlin et al. (30) who stated, “Although, shared decision making (SDM) is a best practice approach for decision-making communication about health-related issues, it has not yet been routinely adopted by most health-care professionals” (p. 2).

Looking at this discrepancy, we suggest that both health and social care knowledge and practice are traditionally anchored in the values, principles, and practices that comprise each profession's academic training, despite the fact that this existing knowledge is still infused with a predominantly positivist and traditional medical model of care (31). Within this context, subjective experience and personal meaning are not seen as part of the “medical hegemony” (32) and there is little recognition of users' knowledge (33).

At the outset of the training our students voiced their sense of familiarity with SDM principles based on their previous learning, both in course work and in practice. They articulated their prior expertise and wisdom to practice partnership, empowerment and authentic listening. This expertise had been formed from the building blocks of the helping professions which emphasize collaboration and client involvement. In a similar vein, this view seems to replicate the responses of family physicians following SDM training where “Most of the competencies sounded intuitively obvious to the physicians and close to what they already do or try to do” [(34), p. 329].

Even though the students claim to work with SDM, the first step in “doing” the SDM process requires identifying the existing power hierarchies that exist in professional relationships particularly regarding the role of users' experiential knowledge compared to professional academic knowledge. Here the students tended to attribute far more to listening to their own expertise than to users' voices and experience.

### “We Don't Do It Here”: Pressures and Excuses

Although patient involvement and participation in healthcare decision making has been associated with enhanced users' compliance and improved treatment outcomes, implementation of SDM by medical health practitioners is still rare (35). Consequently, there is little guidance on how to implement SDM in clinical social care practice (36). Students who started to integrate the SDM principles during the training module often reported on barriers that they confronted in the field which mirror findings from the mental health field (21). These barriers were perceived by the students to exist in two areas: the first is inherent in the organizational structure of the care setting whether it be a hospital or a school or a prison, which is grounded in a bureaucracy such as in the development of practice protocols. The second area relates to the cultural environment as characterized by the workers and users themselves.

In order to address the first area, turning to the healthcare literature for guidance, various organizational-level characteristics have been identified that may impact the implementation of SDM. For example, Scholl et al. (37) focus on characteristics such as the extent to which the organization's main purpose and vision for the future supports SDM and the degree to which organization heads proactively support SDM. Also, it depends on the extent to which an organization's culture supports SDM, as well as the degree to which other aspects of service provision conflict or align with SDM. These authors emphasize that many features have also been shown to influence implementation at the system level such as the degree to which SDM is included as a criterion in the accreditation of healthcare institutions, or whether legislation requires the practice of SDM. However, perhaps of relevance to our present discussion is the extent to which the initial and continuing education and licensing of health professions includes genuine SDM training (37).

Furthermore, implementation models can inform us about how to practice in the face of organizational barriers. This includes individual or collective evaluations of the concept and worth of user involvement, the quality of the relationships that exist between the different participants, the organizational environments in which these relationships occur, as well as the autonomy and abilities of the relevant figures involved in facilitating the change process (33). We agree that for significant involvement to occur there needs to be new patient/user definitions of how to address the quality of care relationships. Just as importantly perhaps, future organizational planning should support the time spent with users and be more flexible in meeting their needs (33).

Secondly, the cultural context was addressed by some of the students in both sites who spoke of how their professional colleagues and supervisors had not previously been exposed to discourses on partnership work and SDM. These same students anticipated the barriers they would face as professionals and spoke of the need for a real change such as accepting experiential knowledge as valid knowledge within the profession and not merely feigning “lip service.” Here too, research with physicians had previously identified similar barriers to shared decision making and user involvement among professionals (38). These barriers included conceptual differences in the interpretation and meaning of involvement between service users and professionals, and a professional resistance to sharing or transferring power (33).

Thus, it became clear that, like physicians who had undergone such a training, one training module seemed insufficient to promote lasting change in their perceptions and behavior. “The complexity of the barriers to SDM means that a single educational intervention is unlikely to be effective in changing behavior even among predisposed physicians” [(34), p. 330].

There are also barriers on the side of the users. The students reported on the limited resources that the users were able to access, whether in locating information and other material resources or in the form of personal characteristics such as a cognitive impairment. Furthermore, the accepted norm of “passiveness” has been grounded in a long history of role socialization within the professional relationship and this too hinders the users' engagement. The students pointed to user expectations from professionals to lead and make the decisions. Some students suggested that there is a need to teach users how to become partners, a comment that is borne out in the literature on SDM in mental health (16, 39). In recent studies, the SDM process was linked to users' personal recovery, person-centered care, and engagement in the process (16, 18). In addition, users who were involved in educating professionals developed partnerships with the providers that reflected the users' own priorities (40).

Finally, user involvement in professional education has benefits for both sides. For professionals, to expand their knowledge base and for users to increase their confidence, self-respect, and feelings of empowerment that support their ability to become active partners (40, 41).

### Lessons Learned

This study highlighted the relevance of SDM for both groups of students in the allied health and social care professions. However, it became clear that one training module seemed insufficient to promote lasting change in their perceptions and behavior. We therefore believe that an essential prerequisite to the expansion and promotion of the values and practice of SDM necessarily requires that students are challenged by a variety of critical ideologies and discourses throughout their professional education in a wide variety of areas. These alternative discourses, such as those grounded in critical theory (42, 43) are all but absent in such curricula, as are innovative pedagogic methods that can challenge the existing dominant discourse and perspectives (44). This type of pedagogy could be strengthened by a modeling of the student-teacher interaction that reflects the principles of partnership and collaboration across the various courses in the academic degree. Furthermore, the inclusion of service users throughout their professional education in a variety of roles would expose the students to the value of user knowledge and its role in developing professional relationships.

One of these roles is that of co-teacher. This pedagogy enables the development of partnership and dialogue within the classroom setting which can facilitate the development of an inclusive knowledge base and may address the concerns raised by our students regarding users' disempowerment in the professional relationship. Examples of such a pedagogy have been reported in both social work and occupational therapy in Israel (45, 46) and can be replicated in additional allied health and social care professions. Moreover, following Simmons (47) who reports on the contributions of young people as co-trainers, we believe that a co-teaching pedagogy needs to be incorporated into the ongoing design and development of future SDM training modules.

An important contribution to the effectiveness of SDM implementation into clinical practice is the use of SDM aids. This tool comprises tasks that promote a structured conversation for conveying the complexity of information which helps the user to participate more meaningfully. Thus, aids can increase user self-determination and engagement that effectively supports the decision-making process itself (48, 49) and we suggest that it may support students in their move to practice.

## Conclusions

This module was developed as an initial endeavor to expand the use of SDM into the health and social care professions. This article brings a modest look at the students' experiences, but we suggest that future trainings need to be developed and evaluated in a systematic way.

While the training module was to our knowledge the first to be introduced to students at two sites in the allied health and welfare professionals, they were both conducted in the same geographical region, namely Israel. Therefore, some aspects of the structure of the module and our resulting conclusions may not be applicable to similar academic settings in other countries.

Building on the burgeoning literature on SDM trainings with physicians and other medical professions, we hope that our experience with this pilot training module encourages others to develop additional training modules and thereby further the vision of social justice and an improved implementation of shared understanding and undertaking between service users and providers.

## Data Availability Statement

The raw data supporting the conclusions of this article will be made available by the authors, without undue reservation.

## Author Contributions

CG, AC, and DRA contributed equally to the research and writing of the manuscript. All authors approved the final version.

## Conflict of Interest

The authors declare that the research was conducted in the absence of any commercial or financial relationships that could be construed as a potential conflict of interest.

## Publisher's Note

All claims expressed in this article are solely those of the authors and do not necessarily represent those of their affiliated organizations, or those of the publisher, the editors and the reviewers. Any product that may be evaluated in this article, or claim that may be made by its manufacturer, is not guaranteed or endorsed by the publisher.
